# Identification of an Efflux Transporter LmrB Regulating Stress Response and Extracellular Polysaccharide Synthesis in *Streptococcus mutans*

**DOI:** 10.3389/fmicb.2017.00962

**Published:** 2017-06-08

**Authors:** Jia Liu, Lihong Guo, Jianwei Liu, Jianying Zhang, Huihui Zeng, Yang Ning, Xi Wei

**Affiliations:** ^1^Guangdong Provincial Key Laboratory of Stomatology, Guanghua School of Stomatology, Hospital of Stomatology, Sun Yat-Sen UniversityGuangzhou, China; ^2^Department of Operative Dentistry and Endodontics, Xiangya Stomatological Hospital, Central South UniversityChangsha, China; ^3^Applied Oral Sciences, Faculty of Dentistry, University of Hong KongHong Kong, Hong Kong

**Keywords:** *Streptococcus mutans*, efflux transporter, biofilm, extracellular polysaccharide, stress response

## Abstract

Efflux transporters have been implicated in regulating bacterial virulence properties such as resistance to antibiotics, biofilm formation and colonization. The pathogenicity of *Streptococcus mutans*, the primary etiologic agent of human dental caries, relies on the bacterium’s ability to form biofilms on tooth surface. However, the studies on efflux transporters in *S. mutans* are scare and the function of these transporters remained to be clarified. In this study, we identified an efflux transporter (LmrB) in *S. mutans* through cloning the *lmrB* gene into *Escherichia coli*. Introducing *lmrB* into *E. coli* conferred a multidrug-resistant phenotype and resulted in higher EtBr efflux activity which could be suppressed by efflux inhibitor. To explore whether LmrB was involved in *S. mutans* virulence properties regulation, we constructed the *lmrB* inactivation mutant and examined the phenotypes of the mutant. It was found that LmrB deficiency resulted in increased IPS storage and prolonged acid production. Enhanced biofilm formation characterized by increased extracellular polysaccharides (EPS) production and elevated resistance to hydrogen peroxide and antimicrobials were also observed in *lmrB* mutant. To gain a better understanding of the global role of LmrB, a transcriptome analysis was performed using *lmrB* mutant strain. The expression of 107 genes was up- or down-regulated in the *lmrB* mutant compared with the wild type. Notably, expression of genes in several genomic islands was differentially modulated, such as stress-related GroELS and scnRK, sugar metabolism associated *glg* operons and *msmREFGK* transporter. The results presented here indicate that LmrB plays a vital global role in the regulation of several important virulence properties in *S. mutans*.

## Introduction

As the primary etiologic agent of human dental caries, *Streptococcus mutans* has evolved to be the normal member of oral flora (Smith and Spatafora, 2012). The primary virulence characteristics that allow *S. mutans* to survive and thrive in oral cavity are their ability to form tenacious biofilms and respond to environmental challenges presented by oxidative stress and changes in carbohydrate availability (Cheng et al., 2016). *S. mutans* has evolved a multitude of regulatory strategies to integrate its cellular response to environmental change and control virulence expression, such as two-component signal transduction systems (TCSTS) derived from the investigation of VicRKX, LiaFSR, ScnRK, and ComCDE quorum-sensing (QS) (Smith and Spatafora, 2012). Recently, efflux systems have been reported as another mechanism responsible for bacterial pathogenicity and biofilm development (Piddock, 2006). Efflux pumps are proteins localized on the cytoplasmic membranes, which allow the microorganisms to extrude out the toxic substances like antimicrobial agents, metabolites from the cell and thereby keep their internal environment stable (Soto, 2013). On the basis of structural characteristics, the multidrug efflux systems are classified into several families: the ATP-binding cassette (ABC) superfamily, RND, MFS, SMR, and MATE (Li and Nikaido, 2004). Other than characterized only as drug exporter, evidence of efflux systems involvement in regulating bacterial virulence and resistance has been obtained in various bacterial species. In *Campylobacter jejuni*, RND family efflux CmeABC was essential for its colonization in chickens (Lin et al., 2003). In *Escherichia coli and Salmonella enteric* serovar Typhimurium, efflux transporters were essential for biofilm matrix synthesis and biofilm formation (Soto, 2013). A RND-like efflux transporter in *Pseudomonas aeruginosa* has been implicated in QS regulation through actively transporting QS molecules (Diggle et al., 2002). In spite of extensive studies on the importance of efflux systems in Gram-negative bacteria, the roles of efflux systems in Gram-positive bacteria are rarely elucidated, especially in *S. mutans*. Six efflux pumps have been identified in *Staphylococcus aureus* and the macrolide efflux pump encoding gene *msrA* has been reported to be associated with polymicrobial-biofilm formation (Weigel et al., 2007). In *Listeria monocytogenes*, an ABC transporter was involved in negative regulation of biofilm formation, which exported signaling molecules to activate a pattern of genome expression characteristic of planktonic growth of *L. monocytogenes* (Zhu et al., 2008). It has also been reported that possible efflux pump encoding genes were elevated when *S. mutans* was exposed to heat shock, osmotic stress, and oxidative conditions, suggesting a potential role of efflux system in regulating *S. mutans* virulence (Nagayama et al., 2014). Moreover, the *rcrRPQ* efflux system was shown to be involved in the ability of *S. mutans* to initiate biofilm formation, oxidative and antimicrobials response (Ahn et al., 2014). In the present study, we hypothesized that LmrB was an efflux pump in *S. mutans* based on the bioinformatical analysis. To substantiate this, we cloned and expressed LmrB in *E. coli*. And the exact role of LmrB in *S. mutans* virulence regulation and stress response was investigated through constructing the *lmrB* deletion mutant. The global gene expression upon *lmrB* deletion was profiled to uncover the role of LmrB.

## Materials and Methods

### Bacterial Strains and Culture Conditions

*Streptococcus mutans* UA159 cells were cultured in BHI broth (Difco, Sparks, MD, United States) at 37°C under anaerobic conditions (90% N_2_, 5% CO_2_, and 5% H_2_) and 1% sucrose was included in the medium for biofilm formation. *E. coli* strains were grown in LB medium (Difco, Sparks, MD, United States). Antibiotics and other supplements were used in the following final concentrations: Spe 800 μg/ml; kanamycin 50–100 μg/ml. The plasmid and primers used were provided in Supplementary Table S1. The CSP was synthesized by Life Invitrogen (Shanghai, China).

### Construction of Recombinant Plasmid Carrying LmrB for *In Vivo* Studies

The *S. mutans lmrB* gene was amplified from genomic DNA using the polymerase chain reaction (PCR) system (TaKaRa) with appropriate primers (Supplementary Table S1). The amplicon was cloned in the pET28a expression vector to create the construct pET 28a-*lmrB* with His-tag. The resulting recombinant pET28a-*lmrB* with His-tag was sequenced for confirmation and transformed into the *E. coli* BL21 (DE3) cells to assess the *in vivo* role of LmrB in *E. coli*.

Localization of LmrB in *E. coli* was determined as previously described with modifications (Yeh et al., 2013). In brief, *E. coli* BL21 (DE3) cells harboring recombinant plasmid were cultured at 16°C with the addition of 0.3 mM isopropyl-β-D-thiogalactopyranoside to induce protein expression. After a further incubation for 4 h, the cells was collected, and then broken by sonication and ultracentrifuged at 100,000 × *g* for 60 min. The resulting pellet was re-suspended in buffer [10 mM Tris-HCl (pH 7.3), 5 mM MgCl_2_, and 2% *N*-Lauroyl Sarcosine Sodium] and ultracentrifuged again. The total protein (the sonicated cell suspension) and the membrane protein (pellet from the second ultracentrifugation) were separated by SDS–PAGE and probed with an anti-His antibody. The proteins of *E. coli* cells transformed with pET28a were used as control.

### Ethidium Bromide (EtBr) Efflux Assay

Standard EtBr efflux assay was used to study the efflux activity of *E. coli* cells with pET28a-*lmrB* (Rodrigues et al., 2008). Cells were grown to an OD_600 nm_ of 0.6 and 200 μL of cells was loaded into 96-well black plates and mixed with EtBr for 30 min for EtBr accumulation. The EtBr-loaded cells were washed and re-suspended in PBS with or without the efflux pump blocker, carbonyl cyanide m-chlorophenyl hydrazone (CCCP) (100 μM), added at 10 min (Bansal et al., 2016). The fluorescence was determined by a microplate reader (Promega GloMax, United States) at excitation and emission wavelengths of 525 and 605 nm. The obtained raw data were normalized to the EtBr-loaded cells, which was regarded as relative fluorescence equivalent to 1. In all the cases, *E. coli* BL21 (DE3) with pET28a vector was used as blank control.

### Determination of Minimum Inhibitory Concentrations (MIC)

Susceptibilities of *E. coli* BL21 (DE3) containing pET28a-*lmrB* against different groups of antibiotics including ampicillin, chlorhexidine (CHX), ofloxacin, and chemical agent like EtBr were carried out in 96-well polystyrene plates using a microdilution method as described previously (Xu et al., 2010). Bacterial growth was monitored after 24 h of incubation at 37°C and the MIC was defined as the lowest concentration of the antimicrobials that inhibited visible growth.

### Sequence Analysis of Putative Efflux Pump Protein LmrB

The amino acid sequence of LmrB was obtained from National Center for Biotechnology Information (NCBI) (NP_721163.1). The number of transmembrane domains was predicted by TMHMM server^1^, and the secondary structure of the protein was determined using PREDICT PROTEIN (Rost et al., 2004). In addition, a 3D model of LmrB was built (*E. coli* YajR transporter was used as template) by SWIISS-MODEL using standard procedure and was analyzed.

### Construction of *lmrB* Mutant and the Complemented Strain

The genomic nucleotide sequence encoding *lmrB* was obtained from NCBI GenBank (*lmrB* locus tag, SMU_745). Two 480 bp fragments containing regions of DNA upstream and downstream of *lmrB* were amplified from a genomic DNA template using the primers *lmrB* LF and *lmrB* RF listed in **Table 1**. The left flank region was digested with Xho I and Hind III, then ligated to MCS I of pFW5, which was digested similarly, generating plasmid pFW5:LF. The right flank region was then inserted into the MCS II of pFW5:LF using Pst I and Spe I, creating plasmid pFW5-LF:RF. The pFW5-LF:RF gene replacement vector was transformed into *S. mutans* UA159 in the presence of CSP. Transformants were selected on BHI agar plate containing 800 μg/ml Spe and confirmed by PCR and sequencing. Complementation construct containing the *lmrB* ORFs with the putative promoters were subcloned into the MCSI sites of pFW 5 to create pFW 5-*lmrB*com. The construct was naturally transformed into *lmrB* mutant. Transformants without desired antibiotic resistance profiles were screened for complementation by PCR. The confirmed complemented strains were named pFW 5-*lmrB*com.

**Table 1:** Antibiotic sensitivities (MIC) of *Escherichia coli* transformed with pET28a-*lmrB.*

Antimicrobials	Bacterial strains		
	
	*E. coli BLll*	*E. coli pET28a-lmrB*	*E. coli* pET28a
Ampicillin	2	2	2
Chlorhexidine	0.8	3.2	0.8
Ofloxacin	0.1	0.4	0.05
EB	250	500	250


### Transformation Assay

To assess the effect of *lmrB* deletion on competence development, we examined the transformation of *S. mutans* upon *lmrB* deletion using the plasmid PVA838 (Moye et al., 2016). In brief, *S. mutans* UA159 and its derivatives grown to mid-logarithmic phase (OD_600_ = 0.5) were diluted 1:100 in fresh BHI medium. Cultures were grown to an OD_600_ of 0.1, and 1 μM of CSP and 100 ng of the plasmid PVA838 were added to cultures. After 3 h of growth, cultures were diluted and plated on BHI agar and on BHI agar containing erythromycin (10 μg/ml). After 24–48 h of incubation at 37°C in a 5% CO_2_ aerobic atmosphere, CFUs were enumerated. Transformation efficiency is expressed as the number of erythromycin resistant colonies divided by the total number of viable colonies multiplied by 100.

### Intracellular Polysaccharide Determination

The IPS content of bacterial cultures was determined as described (Busuioc et al., 2009). In brief, bacterial cultures were heated for 5 min at 100°C and bacteria were collected by centrifugation at 4,000 × *g* for 10 min, followed with washing twice with ice-cold water. Bacterial suspension was used for dry weight and IPS determination. The IPS was extracted with 5.3 M KOH and quantified using iodine solution [0.2% (w/v) iodine in 2.0% (w/v) potassium iodide solution] and glycogen was used as standard. The amount of IPS was normalized to the dry weight of bacteria.

### Glycolytic pH Drop Assay

The ability of *S. mutans* strains to undergo glycolysis in an increasingly acidic environment was evaluated by pH drop experiments with slight modifications to the previously published method (Cheng et al., 2014). *S. mutans* strains grown to mid-logarithmic phase (OD_600_ = 0.5) were harvested by centrifugation (4,000 × *g*, 4°C, 10 min), washed with 0.5 mM potassium phosphate buffer containing 37.5 mM KCl and 1.25 mM MgCl_2_ (pH 7.0) and re-suspended in 10% culture volume of the same solution. Glycolysis was initiated by the addition of glucose to give a final concentration of 1% (wt/vol). The resulting decrease in pH, as a result of *S. mutans* glycolytic activity, was monitored (Corning pH meter 240; Corning, NY, United States). To study differences in sugar storage, changes in pH were also monitored when cells were re-suspended in salt solution without addition of glucose.

### Assay of Oxidative Stress Tolerance

*Streptococcus mutans* strains grown to mid-logarithmic phase (OD_600_ = 0.5) were harvested by centrifugation and re-suspended in 5 ml 0.1 M glycine buffer (pH 7.0). Hydrogen peroxide (H_2_O_2_, Fisher Scientific, Fair Lawn, NJ, United States) was then added to give a final concentration of 0.02% (vol/vol) for oxidative stress tolerance assays. Catalase (10–25 kUnits/ml; Sigma) was added to inactivate the H_2_O_2_ after incubation for 15, 30, or 45 min. The culture was serially diluted in 0.1 M glycine buffer (pH 7.0), and the survival rate was determined by plating in triplicate on BHI plates (Cheng et al., 2016).

To further confirm the role of LmrB in oxidative stress tolerance, *S. mutans* was collected for RNA extraction after 2 h exposure to 0.02% H_2_O_2_ and cDNA synthesis was performed with a SuperScript cDNA Synthesis kit (Invitrogen). The *lmrB* expression under oxidative conditions was determined using qRT-PCR. 16S rRNA was used as the internal control with the following reaction program: 95°C for 2 min, followed by 40 cycles of 95°C for 15 s and 60°C for 30 s. Threshold cycle (CT) values were determined and data were analyzed with the 2^-ΔΔCT^ method.

### Biofilm Formation and Structure Imaging

*Streptococcus mutans* UA159 strains grown to mid-logarithmic phase (OD_600_ = 0.5) were diluted 1:100 in BHI containing 1% sucrose. For biofilm formation, aliquots (3 ml) of *S. mutans* strains in BHI-1% sucrose were placed into a 24-well plate containing polystyrene blocks and cultured at 37°C anaerobically for 24 h allowing biofilm formation. To determine the biomass and analyze structure alteration, SYTO 9 (Molecular Probes, Invitrogen, Carlsbad, CA, United States) was employed to label bacterial cells. As the main component of biofilm, exopolysaccharides were labeled with Alexa Fluor 647-labeled dextran conjugate (Molecular Probes) as previously described (Koo et al., 2010). Images were obtained with CLSM (LSM 710, Carl Zeiss, Germany) with a 20× objective and image series were generated by optical sectioning at each position. The three dimensional re-construction of biofilms was performed and the biomass of EPS and bacteria was analyzed using COMSTAT software.

### Antimicrobial Susceptibility Test

The antimicrobial susceptibility of *S. mutans* strains was determined by the microdilution method as described previously (Xu et al., 2010). Briefly, 0.1 ml of *S. mutans* cells (OD_600_ = 0.6) was added into 96-well plate that contained 0.1 ml of various antibiotics (CHX, penicillin, and erythromycin) at concentrations prepared from twofold serial dilutions. After 12 h of anaerobic growth at 37°C, bacteria were plated onto BHI agar plates and observed after overnight growth.

For biofilm formation abilities of *S. mutans* strains under antimicrobial pressure, *S. mutans* cells (OD_600_ = 0.6) were diluted 1:100 in fresh BHI-1% sucrose with sub-inhibitory concentrations (sub-MIC) (0.25xMIC) of chlorhexidine for biofilm formation in flat-bottom 96-well microtiter plates (Corning Inc.) After 12 and 24 h of incubation, the biofilms were quantified using crystal violet staining.

### Transcriptome Analysis

RNA samples from the wild type and the *lmrB* mutant were extracted as follows: cells were grown to mid-logarithmic phase (OD_600_ = 0.5) and collected by centrifugation. The obtained *S. mutans* cells were suspended in lysis buffer (30 mM Tris-HCl, 1 mM EDTA, 20 mg/mL lysozyme, [pH8.0]) and incubated at 37°C with gentle agitation for 30 min. Total RNA was purified using RNeasy Mini kits (Qiagen) according to the manufacturer’s instructions. Extracted RNA with purity (A260/A280) more than 2.0 was subjected to microarray preparation based on the manufacturer’s standard protocols. Briefly, 1 μg of total RNA from each sample was amplified and transcribed into fluorescent cRNA. The labeled cRNAs were hybridized onto the Whole Genome Oligo Array (4x44K, Agilent Technologies) and the arrays were scanned by the Agilent Scanner G2505B. Subsequent data processing was performed using the GeneSpring GX v11.5.1 software package (Agilent Technologies). Microarray data have been deposited at NCBI-GEO (accession no. GSE98374). DEGs with statistical significance were analyzed for protein–protein interactions (PPIs) through Cytoscape software (Institute of Systems Biology, Seattle, WA, United States) and functionally annotated through GO and Database for Annotation, Visualization and Integrated Discovery (DAVID) version 6.7. The expression of selected genes was validated by qRT-PCR with gene-specific primers shown in Supplementary Table S1.

### Statistical Analysis

Data analyses were performed using SPSS 13.0 and an exploratory data analysis was performed to check the assumptions of the equality of the variances and the normal distribution of errors. The data were analyzed using ANOVA, followed by Tukey’s multiple comparison tests and considered statistically significant at *P* < 0.05.

## Results

### LmrB Exhibited Efflux Pump-Like Behavior

**FIGURE 1:**
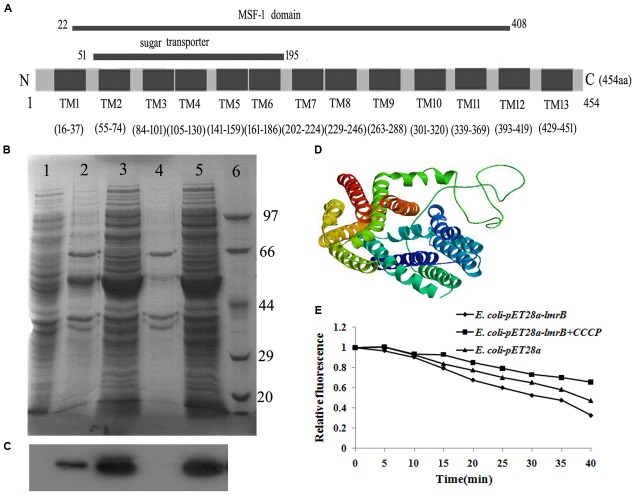
*lmrB* in *Streptococcus mutans* UA159 encoded an efflux transporter. **(A)** Domain architecture of LmrB. The conserved domain in the N-terminus of LmrB was found by searching CDD database on the National Center for Biotechnology Information (NCBI) website. The protein contains 13 transmembrane (TM) regions, a conserved sugar transporter domain and a MFS motif. **(B)** Separation of total cell lysate and isolated membrane proteins from *Escherichia coli* transformed with pET28a-*lmrB* with His-tag using SDS–PAGE profile. Lane 1: total protein of control group (*E. coli* cells transformed with pET28a); lane 2: membrane protein of *E. coli* transformed with pET28a–*lmrB*-His-tag; lanes 3 and 5: total protein of *E. coli* transformed with pET28a–*lmrB*-His-tag; lane 4: membrane protein of control group (*E. coli* cells transformed with pET28a). **(C)** Proteins were transferred to nitrocellulose membrane and probed with anti-His tag. The Western-blot analysis further confirmed that the recombinant protein was integrated into the membrane of *E. coli*. **(D)** The 3D model of LmrB using *E. coli* YajR transporter as template. **(E)** Fluorometric EtBr efflux assay. *E. coli* with pET28a-*lmrB* exhibited higher efflux activity characterized by the low fluorescence intensity.

*lmrB* encoded a putative ATP-type multidrug resistance transporter (454 aa). The putative gene product was membrane associated, with 13 predicted transmembrane domains and the N-terminal region contained a MFS domain and a sugar transport domain (**Figure 1A**). To make sure that this protein could be expressed in correct form and could successfully locate on the membrane of *E. coli* cells, LmrB with His-tag was expressed in *E. coli* using pET28a. The total protein and the membrane protein of *E. coli* with and without isopropyl-β-D-thiogalactopyran -oside induction were isolated and probed with anti-His antibody. Using Western-blot analysis, bands corresponding to the expected size were identified in the total protein (**Figure 1B**, lanes 3 and 5) and membrane protein (**Figure 1B**, lane 2) of *E. coli* transformed with pET28a–*lmrB*-His-tag, while no band with the expected size was observed in the total protein (**Figure 1B**, lane 1) and membrane protein (**Figure 1B**, lane 4) of control group (*E. coli* cells transformed with pET28a). Notably, the band in the total protein was darker than that in the membrane protein, which suggested that only part of the recombinant protein was integrated into the *E. coli* cell membrane. The Western-blot analysis further confirmed that the recombinant protein was integrated into the membrane of *E. coli* (**Figure 1C**)

The structure topology of LmrB resembled *E. coli* YajR transporter, which belonged to MFS transporter (**Figure 1D**). Secondary structure analysis revealed 75.33% alpha-helix, 1.76% beta-sheet, and 22.91% loop in LmrB.

To assess whether LmrB possessed efflux activity, we examined the EtBr efflux activity of *E. coli* upon *in trans* expression of LmrB. As demonstrated in **Figure 1E**, the efflux activity of E. *coli* was higher for the bacterial culture harboring cloned pET28a-*lmrB* characterized by the low fluorescence intensity. And the EtBr efflux was suppressed with the addition of the efflux pump blocker CCCP. The results suggested that LmrB possessed efflux activity.

### LmrB Expression Conferred Antimicrobial Resistance on *E. coli*

To confirm the function of LmrB as a potential multidrug efflux pump, we functionally expressed the candidate multidrug efflux pump in *E. coli* and examined the changes in the antimicrobials sensitivity. As listed in **Table 1**, a rise in the MIC values of CHX and ofloxacin was observed upon ectopic expression of *LmrB* in *E. coli* as compared with the control, with fourfold and eightfold increase in MIC, respectively. An increase in the MIC of EtBr was also noted. These results indicated that upon expression of LmrB, a multidrug resistance nature was conferred to *E. coli* cells. To further prove that the resistance displayed was due to efflux pump activity, we assessed the sensitivity of *E. coli* against ampicillin, an antibiotic targeted extracellularly. As expect, no change in MIC against ampicillin was observed in *E. coli* upon expression of LmrB.

### *lmrB* Inactivation Resulted in Enhanced IPS Accumulation

**FIGURE 2:**
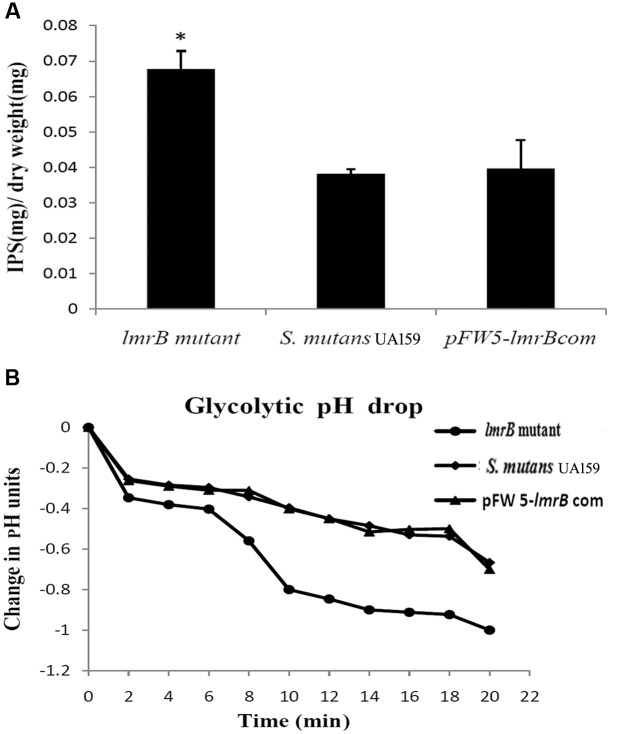
*lmrB* in *S. mutans* UA159 was involved in IPS accumulation and glycolytic pH drop. **(A)** IPS storage in *S. mutans* UA159 strains. More IPS storage in *lmrB* mutant was observed compared with the wild type and the complemented strains. ^∗^Significant difference compared with the wild type (*P* < 0.05). **(B)** Glycolytic pH drop of *S. mutans* UA159 strains in the absence of exogenous glucose. The *lmrB* mutant was able to reduce the pH to lower values compared with the wild type.

In order to investigate the physiological function of LmrB in *S. mutans*, we constructed *lmrB* mutant and pFW5-*lmrB* complemented strains. The mutant and complementary strains were verified by PCR (Supplementary Figure S1). In view of the roles IPS played in supporting *S. mutans* persistence in batch cultures, we assessed the effect of *lmrB* inactivation on *S. mutans* capacity to store IPS. As demonstrated in **Figure 2A**, *lmrB* mutant have significantly higher amounts of IPS compared to the wild type. IPS utilization has been demonstrated to prolong acid production (Busuioc et al., 2009). We monitored the pH change with and without the addition of exogenous glucose to determine the ability of the *lmrB* mutant to reduce the extracellular pH through glycolysis. With the addition of glucose to cells re-suspended in buffer, no difference in kinetics and final pH was observed in the *lmrB* mutant and the wild type (data not shown). When no exogenous glucose was added, any drop in the extracellular pH could be attributed to IPS utilization. The *lmrB* mutant was found to be able to reduce the pH to lower values compared with the wild type in the absence of exogenous glucose (**Figure 2B**).

### *lmrB* Inactivation Resulted in Enhanced Oxidative Stress Resistance

**FIGURE 3:**
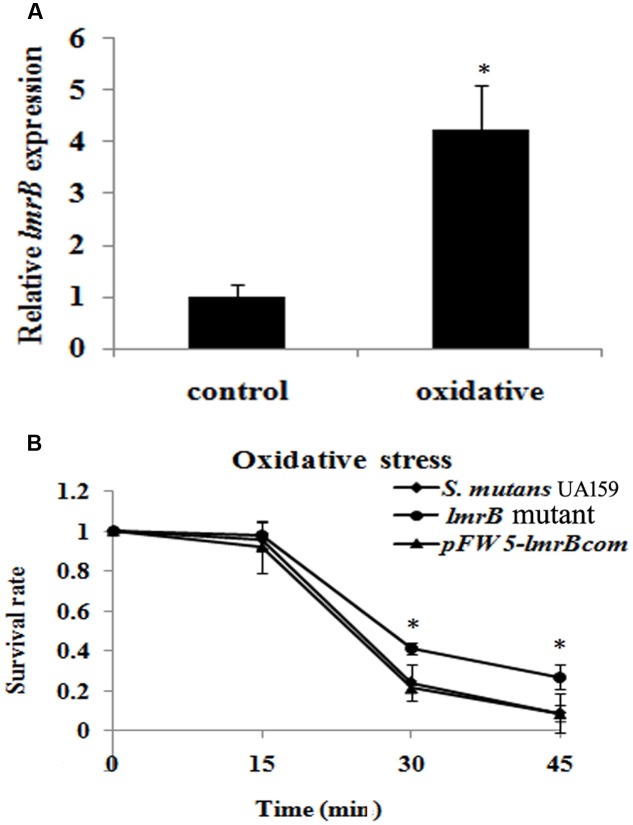
*lmrB* gene in *S. mutans* UA159 was involved in response to oxidative stress. **(A)** Relative expression level of *lmrB* under oxidative conditions. *lmrB* expression was normalized to the levels of 16S rRNA gene, and the fold of expression change was calculated. ^∗^Represented that oxidative stress induced the expression of *lmrB* significantly (*p* < 0.05). **(B)** Oxidative stress assays. H_2_O_2_ sensitivity of *S. mutans* UA159 strains was determined by measuring the survival rate of *S. mutans* UA159 cells at different time points. ^∗^Represented that the *lmrB* mutant was more resistant to H_2_O_2_ killing compared with the wild type and complemented strains (*p* < 0.05).

In order to identify whether LmrB was involved in the environment stress response, we measured the expression levels of *lmrB* under stress condition. It was found that the expression level of *lmrB* was enhanced by 4.23-fold when exposed to oxidative stress (*p* < 0.05) (**Figure 3A**). The involvement of LmrB in oxidative stress response was further supported by the H_2_O_2_ sensitivity assay, which showed significantly increased survival rate in *lmrB* mutant compared with the parental and complemented strains (*p* < 0.05) (**Figure 3B**). The result indicated that *lmrB* inactivation resulted in enhanced resistance to H_2_O_2_ in *S. mutans*.

### Inactivation of *lmrB* Exhibited No Effect on Competence Development

The transformation efficiency of *lmrB* mutant was assessed without the addition of exogenous CSP. In the absence of CSP, we were able to obtain almost the same transformant colonies in the *lmrB* mutant and *S. mutans* (*p* > 0.05). Supplementary Figure S2 showed a typical example of the number of transformants obtained for each strain at the same serial dilution.

### The *lmrB* Mutant Exhibited Enhanced EPS Production

**FIGURE 4:**
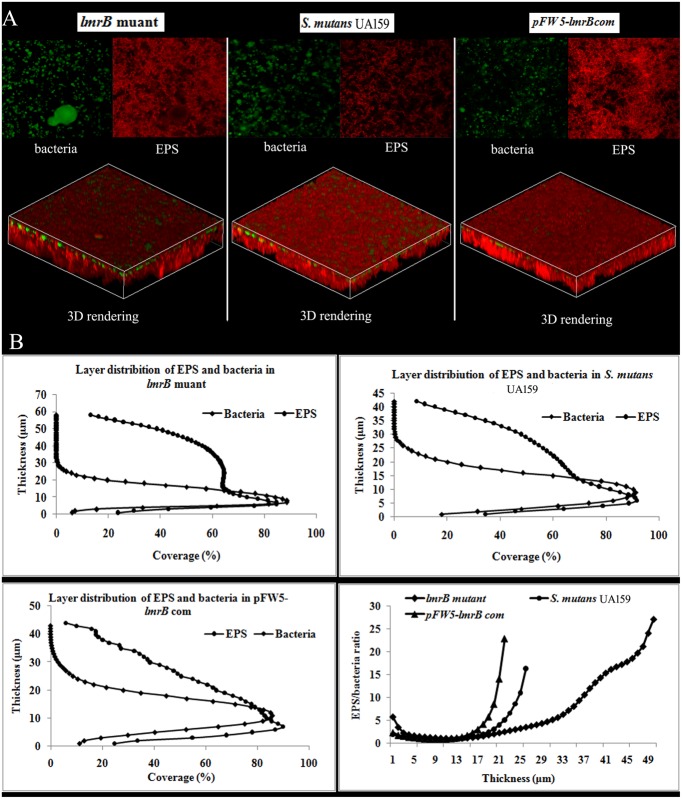
Biofilm architecture and EPS distribution of *S. mutans* UA159 strains. **(A)** Representative double-labeling of 24 h *S. mutans* UA159 strains biofilms. Green, bacteria (SYTO 9); red, EPS (Alexa Fluor 647). **(B)** The distributions of EPS and bacteria at each layer. Quantification of bacteria/EPS biomass was performed with COMSTAT and the ratio of EPS to bacteria at different layers was also quantified.

Since efflux pump was reported to closely associate with biofilm formation in many bacterial species, we explored the role of *lmrB* in biofilm formation of *S. mutans* using CLSM. It was found that the biofilm formed by *lmrB* mutant was significantly thicker (58.0 μm) than those formed by its wild type strain (34 μm) and the complemented strain (32 μm) (*p* < 0.05) (**Figure 4A**). Moreover, the EPS/bacteria ratio on the surface (thickness = 1–5 μm) and at the bottom (height = 47–50 μm) was significantly higher than that of the biofilms formed by the wild type and complemented strains (*p* < 0.05) (**Figure 4B**).

### Inactivation of *lmrB* Altered Antimicrobial Susceptibility and Biofilm Formation Ability

**FIGURE 5:**
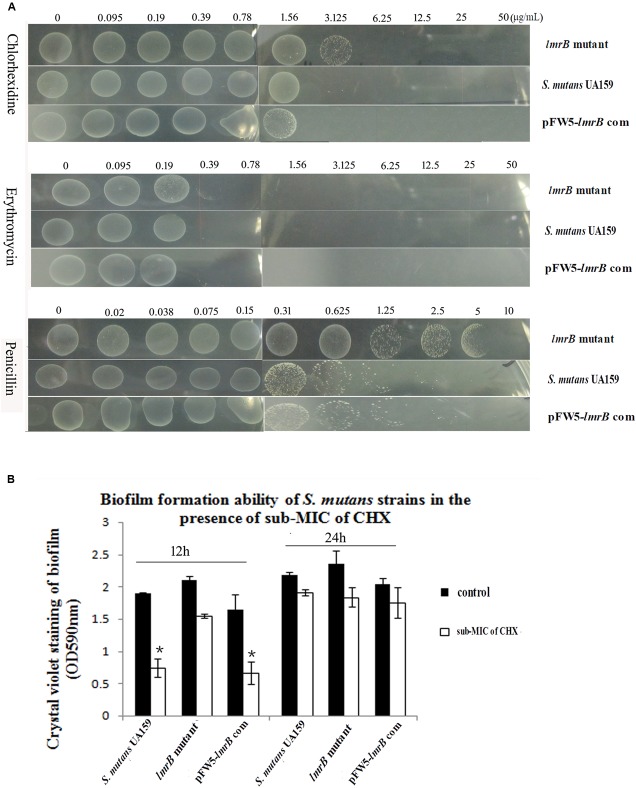
Susceptibilities of *S. mutans* UA159 strains to different antimicrobials. **(A)** Growth of *S. mutans* UA159 and *lmrB* mutant in the presence of different concentration of chlorhexidine (CHX), penicillin, and erythromycin. An overall decreased sensitivity to all the three antimicrobials was observed. **(B)** Biofilm formation ability of *S. mutans* UA159 strains under 0.25xMIC of CHX pressure. No significant difference was observed in the *lmrB* mutant with and without 0.25xMIC of CHX (*p* > 0.05), while 0.25xMIC of CHX suppressed biofilm formation ability of *S. mutans* UA159 in the initial 12 h (*p* < 0.05). ^∗^Significant difference compared with the untreated control (*p* < 0.05).

As previously reported, the inactivation of efflux pump might lead to multidrug hypersusceptibility. In order to explore how *lmrB* affected *S. mutans* antimicrobial sensitivity, we examined the susceptibility of the mutant to different species of antimicrobials. Unexpectedly, the *lmrB* mutant strain showed an overall decreased sensitivity to the treatment of different antimicrobials, especially the antimicrobial targeting bacterial cell wall (**Figure 5A**).

The abilities of *S. mutans* UA159 and the *lmrB* mutant to form biofilm under antimicrobial pressure were also examined. It was found that addition of 0.25xMIC of CHX to the medium differentially affected the ability of the *lmrB* mutant and the wild type to form biofilm in a time-dependent manner. As determined by the crystal violet assay, 0.25xMIC of CHX caused 26.3% reduction in 12 h biofilm of *lmrB* mutant (*p* > 0.05). For the wild type, the 12 h biofilm formation decreased 53.2% compared with that without CHX exposure (**Figure 5B**) (*p* < 0.05). However, after 24 h culture, the biomass of both the mutant and *S. mutans* UA159 biofilms returned to those of without CHX exposure.

### Inactivation of *lmrB* Affected the Transcriptional Profile of Genes for Stress Response and Transporter Activity

To gain better insight into the global role of LmrB and how it relates to physiology and virulence in *S. mutans*, global gene expression was performed by comparative DNA microarray analysis. As shown in Supplementary Tables S2, S3, 107 genes were detected as differentially expressed, among which 66 genes were up-regulated and 41 genes were down-regulated. Based on the NCBI *S. mutans* genome annotation, 58 genes had assigned function whereas 49 genes were hypothetical proteins.

**FIGURE 6:**
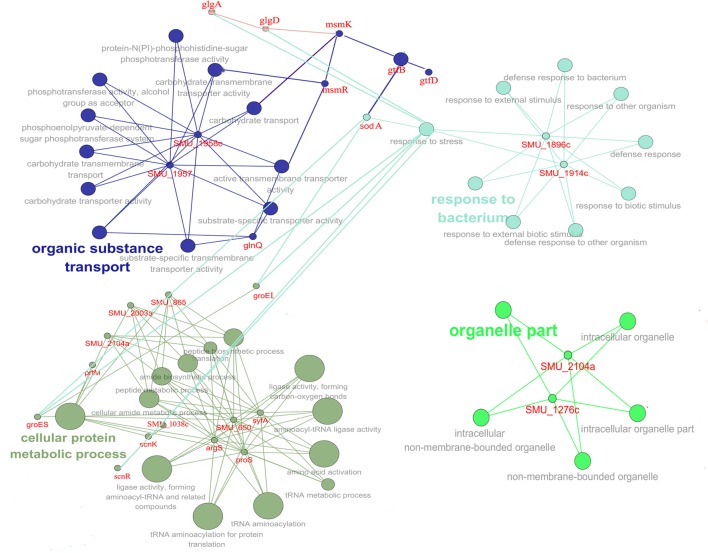
Protein network analysis of the DEGs (*lmrB* mutant versus *S. mutans* UA159 wild type). The interaction data was obtained from the STRING database and interaction network was analyzed by local CYTOSCAPE software. Four protein interaction networks were identified as indicated by different colors.

**FIGURE 7:**
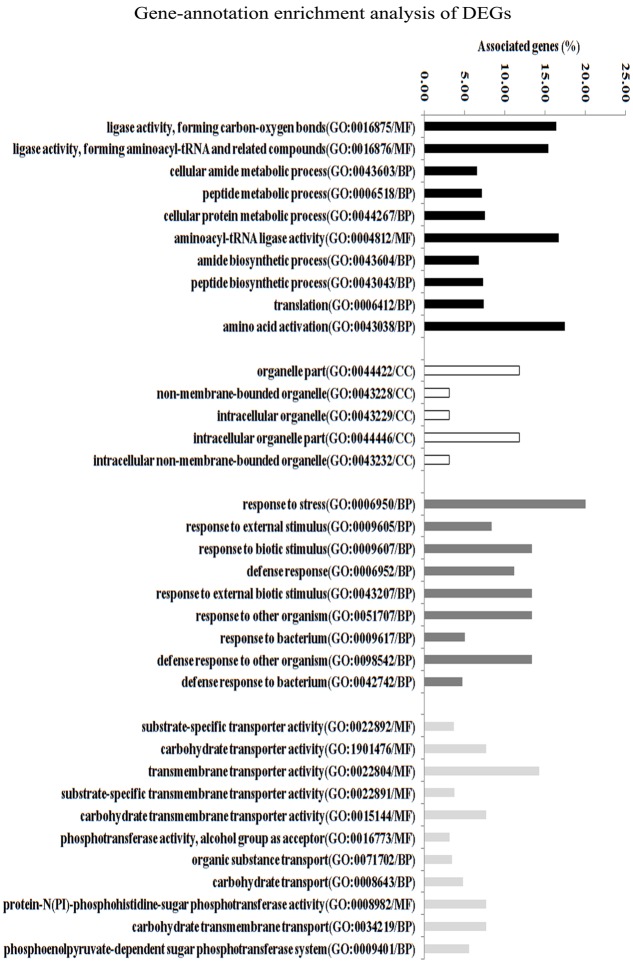
Transcriptome analysis of *S. mutans* UA159 strains (*lmrB* mutant versus *S. mutans* UA159 wild type). Gene ontology enrichment analysis of the DEGs was obtained based on protein network analysis. Four interaction networks represented by different colors were identified among 107 DEGs. The DEGs in GO term response to stress and in GO term TM transporter activity reached 20.6 and 15.8%; GO, gene ontology; BP, biological process; MF, molecular function; CC, cellular component.

In order to gain insights into the biological significance of LmrB in *S. mutans*, we predicted and analyzed interactions among the identified DEGs. The DEGs were clustered in four interaction networks, including metabolic process, organelle part, transportation of carbohydrate and specific substrate and stress response (**Figure 6**). Among them, the three identified networks, cellular protein metabolic process, transportation of carbohydrate and specific substrate and stress response, were closely intertwined. Gene annotation enrichment and functional annotation clustering analysis were then conducted based on the identified protein networks. As shown in **Figure 7**, 20% and 14.23% of the genes related to response to stress and transmembrane transporter activity were affected by the *lmrB* inactivation. Moreover, starch and sucrose metabolism KEGG pathway was identified. Notably, *lmrB* deletion induced the expression of a set of functionally associated genes, like *groES-groEL* molecular chaperones, *glgB-glgC-glgD-glgA-glgP*, two-component system (TCS) s*cnRK* and *msmREFGK.*

To validate the reliability of microarray data, we examined the expression levels of randomly selected genes using qRT-PCR, including up-regulated, down-regulated, and unchanged genes. As shown in Supplementary Figure S3, all genes selected for validation showed a similar expression trend in the qRT-PCR and microarray analysis. These results suggested a good concordance between microarray and qRT-PCR data.

## Discussion

It has been proposed that efflux pumps play vital roles in physiological properties and virulence of various bacterial species (Piddock, 2006). The general reported response of bacteria to efflux pump inactivation included compromised biofilm formation ability, hypersusceptibility to a variety of antimicrobial agents and persistence of bacterium in its ecological niche (Baugh et al., 2012). An increased understanding of the regulation mechanism of efflux pump in virulence expression would be beneficial for combating microbial associated infections. In this study, we confirmed and characterized the potential efflux transporter LmrB in *S. mutans* by cloning and expressing the gene in *E. coli*. The resembling of LmrB structure to the YajR transporter suggested it as a potential member of MFS (Jiang et al., 2013). The protein could successfully locate on the membrane of *E. coli* cells, which further confirmed that the *S. mutans* LmrB protein could function normally as an efflux pump. Transporters of MFS usually function as single-component pumps with 12–14 transmembrane domains, responsible for the transport of sugars, anions, metabolites, and drugs (Li and Nikaido, 2009). Reported substrates of MFS efflux transporter include nalidixic acid, thiolactomycin, ethidium, and the efflux are driven by the electrochemical gradient, typically proton-motive force (Li and Nikaido, 2009). Thus we selected (CCCP), a proton conductor, as the efflux inhibitor, to assess the efflux activity of LmrB. The results of efflux activity assay together with the antimicrobial susceptibility testing data suggested that LmrB may function as an efflux pump protein. Therefore, we construct the *lmrB* deletion mutant to analyze the physiological function of LmrB in *S. mutans*.

*Streptococcus mutans* faces continual environmental challenges in the oral cavity, and therefore the ability to form biofilm and to tolerate environmental insults is critical to *S. mutans’* survival in dental plaque and pathogenesis (Cheng et al., 2016). Sugar metabolism is paramount for the behavior and survival of *S. mutans*, which is fermented by glycolysis to provide energy and produce organic acids to prolong the period of low pH in dental plaque (Colby and Russell, 1997). When there is excess sugar available, IPS is formed and can be utilized as a source of carbohydrate upon nutrient depletion (Gibbons and Socransky, 1962) and prolonged acid production (Busuioc et al., 2009). Since LmrB possessed a sugar transporter domain, we proposed that the deletion of *lmrB* might affect the sugar utilization of *S. mutans*. The microarray identified a number of genes involved in sugar uptake and metabolism as being up-regulated in the *lmrB* mutant. Among them, the *msmEFGK* genomic island is required for the uptake of various disaccharides and/or oligosaccharides substrates and the transcription of the genes could be induced by the carbohydrates (Webb et al., 2008). The other differentially expressed genomic island associated with sugar metabolism was the *glg* operons composed of *glgB-glgC-glgD-glgA-glgP, which* are responsible for the biosynthesis of IPS. *glgA* and *glgB* encodes glycogen synthase and glucan branching enzyme, and *glgC* and *glgD* encode subunits of ADP-glucose pyrophosphorylase (EC 2.7.7.27) (Busuioc et al., 2009). Spatafora et al. (1995) reported that increased cariogenic potential of *S. mutans* was due to constitutive expression of genes encoding glycogen biosynthesis. The constitutive expression of *glg* locus in *S. mutans* was growth phase dependent and was modulated by carbohydrates internalized via the non-phosphoenolpyruvate phosphotransferase system (non-PTS) (Spatafora et al., 1999). The multiple-sugar metabolism (*msm*) operon in *S. mutans*, which provides a pathway for the uptake of non-PTS sugars, was the known system directed the *glg* locus expression (Tao et al., 1993). Transcriptional analysis also revealed differential expression of several genomic islands involving in *S. mutans* stress response. GroELS are heat shock proteins, the production of which is a central feature of bacterial stress responses (Lemos et al., 2001, 2007). The TCS scnRK is important in counteracting oxidative stress in *S. mutans* (Chen et al., 2008). Considering the closely intertwined protein networks among stress response, metabolic process and carbohydrate transportation, *S. mutans* has most likely developed extensive remodeling of transcriptome in response to *lmrB* inactivation. These complex changes might induce the physiological properties alteration by at least four interconnected ways: (1) *lmrB* deletion might destroy the overall interaction of the transporter in *S. mutans* since efflux ATPase domains of transporter can interact with the alternative transporter complex (Webb et al., 2008), and thus induced the expression of *msm* operon; (2) the induced *msm* operon expression as determined by microarray activated the sugar uptake transport systems (e.g., maltose and maltotriose) (Tao et al., 1993), and subsequently resulted in the up-regulation of *glg* operon to accumulate IPS (Spatafora et al., 1999); (3)the accumulated IPS prolonged acid production, which activated the transcription of a variety of general stress response associated genes, including *groEL*, *scnRK*, and *sodA*; (4) the combined effects would result in biofilms with increased biomass that tightly adherent to the surface, thereby enhancing *S. mutans* survival/persistence and cariogenicity.

It has been reported that genetically disrupting any of the MDR efflux systems in *Salmonella Typhimurium*, or chemical inactivation of MDR efflux using EIs repressed biofilm formation (Baugh et al., 2012). Deletions of multipartite efflux system genes *acrB, acrE*, and *tolC* of *E. coli* also resulted in significant reductions in biofilm growth (Bay et al., 2016). However, loss of secondary active multidrug resistance transporters that confer overlapping substrate resistance to a broad range of antimicrobials led to similar or enhanced biofilm formation of *E. coli* (Bay et al., 2016). Similarly, enhanced EPS production was noticed in *S. mutans* biofilm upon *lmrB* inactivation. The observed enhanced biofilm formation phenotypes of *lmrB* mutant cannot be caused by polar effect of mutations based on the observation that *lmrB* was a separate transcription unit (Krol et al., 2014), which was in consistent with the notion that MFS members function as single-component pumps in Gram-positive bacteria (Li and Nikaido, 2004). Therefore, it is intriguing how *lmrB* deletion affected the EPS production? Sugar transporter and metabolism are crucial to the biofilm EPS formation. Upon *lmrB* inactivation, expressions of several carbohydrate transporter and efflux regulators encoding genes were up-regulated as revealed by microarray. The data suggested a potential coordinated regulation of efflux and biofilm formation. Efflux pumps and biofilms formation are two efficient mechanisms utilized by various microorganisms to resist the action of antimicrobials (Bansal et al., 2016). Another major finding of this study was that *lmrB* mutant with enhanced EPS formation exhibited similar or enhanced tolerance to one or more antimicrobials tested. The potential explanation for such a counterintuitive phenotype observed may be linked to the expression of other homologous genes to compensate for the function of LmrB. This speculation was verified by the microarray data, which revealed that genes encoding ABC transporters involving in multiple antibiotic resistances were up-regulated in *lmrB* mutant. Thus, it was possible that upon *lmrB* inactivation the *S. mutans* employed other pathways to induce the expression of genes with the same function to ensure the survival of the bacterium. Our speculation was supported by the previously published data which showed that inactivation of one efflux pump genes would alter the expression of homologous pumps and thus did not significantly change the MICs of antibiotics (Blair et al., 2015). Another example of this was that deletion of acrAB in *E. coli* enhanced carbapenemase resistance due to regulation of general diffusion porins OmpF and OmpC (Saw et al., 2016).

Taken together, our data suggest that LmrB from *S. mutans* is a multidrug efflux pump. By using whole-genome microarrays to profile the transcriptomic responses. The dynamic transcriptional alterations of multiple operons and functional genes, together with enhanced biofilm biomass, indicate metabolic adaptations by this bacterium to compensate for inactivation of *lmrB*. The global analysis revealed a broad impact of the *S. mutans* LmrB on *S. mutans* biofilm formation, stress response and antimicrobials susceptibility.

## Author Contributions

Experiments were performed by the following authors: conceived and designed the experiments-JL, JwL, and XW; performed the experiments-LG and HZ; wrote the paper-JwL, JZ, and YN. The manuscript had been reviewed by all authors before submission.

## Conflict of Interest Statement

The authors declare that the research was conducted in the absence of any commercial or financial relationships that could be construed as a potential conflict of interest.

## Footnotes

1 http://www.cbs.dtu.dk/services/TMHMM/

## Abbreviations

CFU: colony forming unit
CLSM: confocal laser scanning microscope
CSP: competence stimulating peptide
DEGs: differentially expressed genes
EPS: extracellular polysaccharides
GO: gene ontology
IPS: intracellular polysaccharide
LB: Luria–Bertani
MATE: multidrug and toxic compound extrusion
MFS: major facilitator superfamily
MIC: minimum inhibitory concentrations
qRT-PCR: quantitative real-time PCR
RND: resistance nodulation cell division
SMR: small multidrug resistance
Spe: spectinomycin
