# Supplementary material for: Identification of an Efflux Transporter LmrB Regulating Stress Response and Extracellular Polysaccharide Synthesis in Streptococcus mutans
Source: Front Microbiol. 2017 Jun 8;8:962. doi: 10.3389/fmicb.2017.00962 (PMC5463993; doi:10.3389/fmicb.2017.00962)
Supplement: Supplementary file 1 [file Data_Sheet_1.docx]

**Table S1.** Bacterial strains, plasmids, and primers used in the study

| **Bacterial strains** | **Major properties** | **Source or reference** |
| --- | --- | --- |
| ***Bacterial strains***  *S. mutans* UA159 | wild-type, serotype c, virulent strain of caries | Laboratory stock |
| *S.mutans* △*lmrB* | UA159 derivative, *△lmrB* (SMU.745), Spe^r^ | This study |
| *E. coli* DH5α | *endA1,hsdR17,supE44* | Takara Biotechnology |
| *Escherichia coli* BL21 | B F^−^ompT gal dcm lon hsdS_B_(r_B_^−^m_B_^−^) rne131 (DE3) | Takara Biotechnology |
|  |  |  |
| **Plasmids** |  |  |
| pFW5 | Cloning vector contains a spectinomycin-resistance marker (*aad9*) | ^1^Podbielski et al., 1996 |
| pFW5:LF^a^ | 480-bp DNA fragment with *lmrB* flanked by upstream sequences cloned into Xho I–Hind III sites of pFW5, Spe^r^ | This study |
| pFW5-LF:RF^a^ | 480-bp DNA fragment with *lmrB* flanked by downstream sequences cloned into Pst I–SpeI sites of pFW5:LF, Spe^r^ | This study |
| pVA838 | a shuttle vector of oral streptococcal origin | Laboratory stock |
| pET28a | Kan^r^ expression vector | Novagen |
| **Primers** | **DNA sequence(s) (5’-3’)** | **Purpose** |
| *lmrB* LF^b^ | GGCTCGAGTGGTATTCTATCAG (Fw), | 480 bp, *lmrB* left flank, |
|  | GGCGCAAGCTTCTGATAATTAAAGC (Rev) |  |
| *lmrB* RF^b^ | GGCTCGAGTGGTATTCTATCAG (Fw)  GGCGCAAGCTTCTGATAATTAAAGC (Rev) | 480 bp, *lmrB* right flank, |
| *lmrB* | GTTCCTAAGGCACCGGCAAA (Fw) | 324 bp, PCR |
|  | CGCCGGTTTTGTGTTACTGC (Rev) |  |
| aad9 | CTGATGTGAGAAGAGCCATTATGGA (Fw) | 168 bp, PCR |
|  | ATGGAGAAGATTCAGCCACTGC (Rev) |  |
| *glgA* | TGACAAGCCACAAGGCTCAA (Fw),  CGGTGCTCTATGCAGATCGT (Rev) | 539bp, qRT-PCR |
| *msmK* | TCCTTCAGGCTGCGGAAAAT (Fw)  CCGCATAGATACCCGCAACT(Rev) | 412 bp, qRT-PCR |
| *gtfB* | TTCTGATCGCGTGGTTGTCA(Fw) AAAGCTCCCCCATACTTGGC(Rev) | 792 bp, qRT-PCR |
| SMU.1896c | AAGCGCCTGTTCCAATCGTA(Fw)  TGGCTGGGACAGTATCTGGA(Rev) | 71 bp, qRT-PCR |
| SMU.984 | CGGGTAATCAGGCAAAAGTTGG(Fw)  ATTGGGGCTGGGCATTTCA(Rev) | 140 bp, qRT-PCR |
| *gtfD* | TGCAAGCGACGGAAAACAAG (Fw)  CTAGCGATACCCCAACGGTC(Rev) | 348 bp, qRT-PCR |
| SMU.1961c | AACAACAGCATTGACTGCCA(Fw)  TGATATTGTCGGGGGAAGCC(Rev) | 121 bp, qRT-PCR |
| SMU.1611c | TGCTGGAAAAGCCCATAGCA(Fw) GTTGCCCCTATTCTGGCACT(Rev) | 100 bp, qRT-PCR |
| *dedA* | TCCTGCTGCAATCACTGTCC(Fw)  ACGGCTGGTTATCGTTTGGT(Rev) | 240 bp, qRT-PCR |
| SMU.1942c | TGGATTGGAAGCCGTTGGAT(Fw) GCGGTTGCTTTTGCCTCAAT(Rev) | 321 bp, qRT-PCR |
| SMU.1226c | ACAAAGGGGTTGGCAGAGTC(Fw)  GAAGCCAAGTTGCGCAATGA(Rev) | 188bp, qRT-PCR |
|  |  |  |
| 16S rRNA | GCGACGATACATAGCCGACCT (Fw) | 103 bp, qRT-PCR |
|  | TCCATTGCCGAAGATTCCCTA (Rev) |  |

^a^ LF, RF: left flank, right flank; consisted of upstream and downstream sequences of *lmrB*, respectively.

All primers were designed with primer-BLAST and obtained from Invitrogen Biotechnology.

**Table S2. List of up-regulated genes in *lmrB* mutant *versus S. mutans* wild strain**

| **Locus tag** | **Gene name** | **NCBI annotation** | **Fold change** |
| --- | --- | --- | --- |
| SMU.1041 |  | putative ABC transporter, ATP-binding protein | 1.61 |
| SMU.1276c |  | septation ring formation regulator EzrA | 1.59 |
| SMU.711 |  | hypothetical protein | 1.56 |
| SMU.629 | *sodA* | putative manganese-type superoxide dismutase, Fe/Mn-SOD | 1.55 |
| SMU.1646c |  | hemolysis inducing protein | 1.53 |
| SMU.1260c |  | hypothetical protein | 1.65 |
| SMU.1261c |  | putative phosphoribosyl-ATP pyrophosphohydrolase | 1.66 |
| SMU.866 |  | hypothetical protein | 1.65 |
| SMU.1687 | *ppaC* | putative manganese-dependent inorganic pyrophosphatase | 1.62 |
| SMU.1942c |  | putative amino acid binding protein | 1.51 |
| SMU.1349 |  | hypothetical protein | 1.60 |
| SMU.831 |  | hypothetical protein | 1.51 |
| SMU.1958c |  | putative PTS system, mannose-specific IIC component | 1.62 |
| SMU.233 | *ilvC* | ketol-acid reductoisomerase | 1.55 |
| SMU.1073 | *fthS* | formate--tetrahydrofolate ligase | 1.62 |
| SMU.1954 | *groEL* | chaperonin GroEL | 2.53 |
| SMU.1814 | *scnK* | putative histidine kinase, ScnK-like protein | 2.47 |
| SMU.796 |  | hypothetical protein | 1.97 |
| SMU.984 |  | hypothetical protein | 2.25 |
| SMU.1815 | *scnR* | response regulator; ScnR-like protein | 1.69 |
| SMU.1361c |  | TetR family transcriptional regulator | 2.25 |
| SMU.800 |  | hypothetical protein | 1.53 |
| SMU.1657c |  | putative nitrogen regulatory protein PII | 1.69 |
| SMU.1519 | *glnQ* | putative amino acid ABC transporter, ATP-binding protein | 2.04 |
| SMU.1776c |  | hypothetical protein | 2.14 |
| SMU.650 | *alaS* | alanyl-tRNA synthetase | 1.77 |
| SMU.1070c |  | hypothetical protein | 2.47 |
| SMU.444 |  | hypothetical protein | 1.70 |
| SMU_1536 | *glgA* | glycogen synthase | 8.61 |
| SMU.966 |  | homoserine kinase | 1.91 |
| SMU.735 |  | hypothetical protein | 1.53 |
| SMU.1914c |  | hypothetical protein | 2.11 |
| SMU.1872c |  | hypothetical protein | 1.52 |
| SMU.88c |  | mechanosensitive ion channel | 2.01 |
| SMU.1955 | *groES* | co-chaperonin GroES | 2.01 |
| SMU.1246c |  | putative transcriptional regulator | 2.91 |
| SMU.1027 |  | putative transcription regulator | 2.64 |
| SMU.2155 |  | hypothetical protein | 2.09 |
| SMU.1004 | *gtfB* | glucosyltransferase-I | 3.67 |
| SMU.840c |  | hypothetical protein | 1.76 |
| SMU.590c |  | putative transposase, fragment | 1.92 |
| SMU.1896c |  | hypothetical protein | 1.55 |
| SMU.1399 |  | hypothetical protein | 2.23 |
| SMU.2151 | *pgsA* | putative phosphotidylglycerophosphate synthase | 2.27 |
| SMU.1957 |  | putative PTS system, mannose-specific IID component | 2.59 |
| SMU.125 |  | hypothetical protein | 1.71 |
| SMU.18 |  | hypothetical protein | 1.56 |
| SMU.167 |  | hypothetical protein | 2.00 |
| SMU.506 |  | putative type II restriction endonuclease | 1.51 |
| SMU.1512 | *syfA* | phenylalanyl-tRNA synthetase subunit alpha | 1.71 |
| SMU.536 | *trpF* | N-(5'-phosphoribosyl)anthranilate isomerase | 1.53 |
| SMU.2098 | *argS* | arginyl-tRNA synthetase | 2.01 |
| SMU.59 | *purB* | adenylosuccinate lyase | 1.55 |
| SMU.1231c |  | hypothetical protein | 1.53 |
| SMU.1378 |  | hypothetical protein | 1.76 |
| SMU.876 | *msmR* | putative MSM operon regulatory protein | 2.30 |
| SMU.1855 |  | hypothetical protein | 2.41 |
| SMU.2096c |  | hypothetical protein | 1.85 |
| SMU.2048 |  | hypothetical protein | 1.53 |
| SMU.744 | *ftsY* | putative cell division protein FtsY; signal recognition particle (docking protein) | 1.53 |
| SMU.882 | *msmk* | sugar ABC transporter ATP-binding protein | 3.57 |
| SMU.1537 | *glgD* | glucose-1-phosphate adenylyltransferase |  |
| SMU.1191 | *pbp1b* | penicillin-binding protein,membrane carboxypeptidase |  |
| SMU.1611c |  | multidrug transporter | 2.52 |
| SMU_1700c |  | membrane protein controling murein hydrolase activity and penicillin tolerance | 3.69 |
| SMU_1038c |  | Response regulator receiver domain | 2.98 |

**Table S3. List of down-regulated genes in *lmrB* mutant *versus S. mutans* wild strain**

| **Locus tag** | **Gene name** | **NCBI annotation** | **Fold change** |
| --- | --- | --- | --- |
| SMU.796 |  | hypothetical protein | 1.64 |
| SMU.984 |  | hypothetical protein | 2.28 |
| SMU.1361c |  | TetR family transcriptional regulator | 2.31 |
| SMU.2160 |  | transmembrane protein | 1.62 |
| SMU.800 |  | hypothetical protein | 2.02 |
| SMU.1783 | *pros* | prolyl-tRNA synthetase | 2.80 |
| SMU.1070c |  | hypothetical protein | 2.31 |
| SMUt25 |  | tRNA-Glu | 5.57 |
| SMU.1872c |  | hypothetical protein | 1.65 |
| SMU.1360c |  | hypothetical protein | 1.58 |
| SMU.1027 |  | putative transcription regulator | 3.03 |
| SMU.2155 |  | hypothetical protein | 2.34 |
| SMU.2104a | *rpmF* | 50S ribosomal protein L32 | 1.70 |
| SMU.840c |  | hypothetical protein | 1.67 |
| SMU.1116c |  | hypothetical protein | 1.54 |
| SMU.1803c |  | hypothetical protein | 1.59 |
| SMU.2096c |  | hypothetical protein | 2.14 |
| SMU.753 |  | hypothetical protein | 1.53 |
| SMU.1041 |  | putative ABC transporter, ATP-binding protein | 1.63 |
| SMU.817 |  | putative amino acid transporter, amino acid-binding protein | 1.61 |
| SMU.1226c |  | hypothetical protein | 1.67 |
| SMU.1961c |  | putative PTS system, sugar-specific enzyme IIA component | 1.59 |
| SMU.1918 | *dedA* | putative membrane-associated protein DedA | 1.62 |
| SMU.711 |  | hypothetical protein | 1.54 |
| SMU.866 |  | hypothetical protein | 1.71 |
| SMU.1719c |  | hypothetical protein | 1.60 |
| SMU.388 |  | putative integral membrane protein; branched-chain amino acid permease | 1.57 |
| SMU.2089 | *mutL* | DNA mismatch repair protein | 1.53 |
| SMU.1732c |  | hypothetical protein | 1.62 |
| SMU.1922 | *dnaB* | putative chromosome replication protein | 1.54 |
| SMU.782 |  | hypothetical protein | 1.50 |
| SMU.1942c |  | putative amino acid binding protein | 1.63 |
| SMU.648 | *prtM* | foldase protein PrsA | 1.53 |
| SMU.1349 |  | hypothetical protein | 1.73 |
| SMU.378 |  | hypothetical protein | 3.74 |
| SMU.831 |  | hypothetical protein | 1.59 |
| SMU.865 | *rpsP* | 30S ribosomal protein S16 | 1.56 |
| SMU.1706 |  | hypothetical protein | 1.67 |
| SMU.1107c |  | hypothetical protein | 1.56 |
| SMU.2003a | *rpmJ* | 50S ribosomal protein L36 | 1.64 |
| SMU.910 | *gtfD* | glucosyltransferase-S | 1.51 |

Figure S1. PCR analysis of *lmrB* mutant and pFW5-*lmrB*com with primers of *lmrB* ORF (1, 2 and 3) and the flank regions (4, 5 and 6). 1and 4: *lmrB* mutant; 2 and 5: p FW5-*lmrB*com; 3 and 6: *S. mutans* WT

M 1 2 3 4 5 6 M


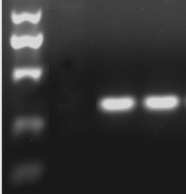

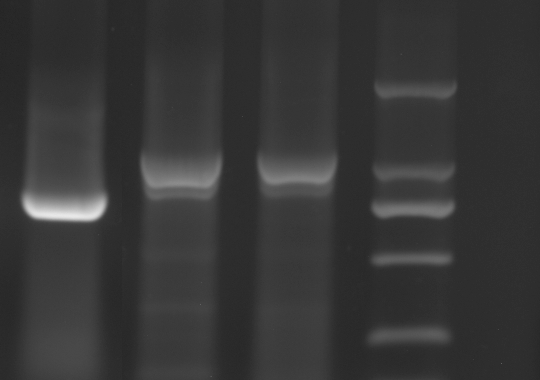


200

500

750

1000

2000

200

100

500

750

1000

Figure S2. Transformation efficiency of *S. mutans* UA159 and *lmrB* mutant. No significant difference in transformation efficiency of *S. mutans* UA159 and *lmrB* mutant was observed (p>0.05)


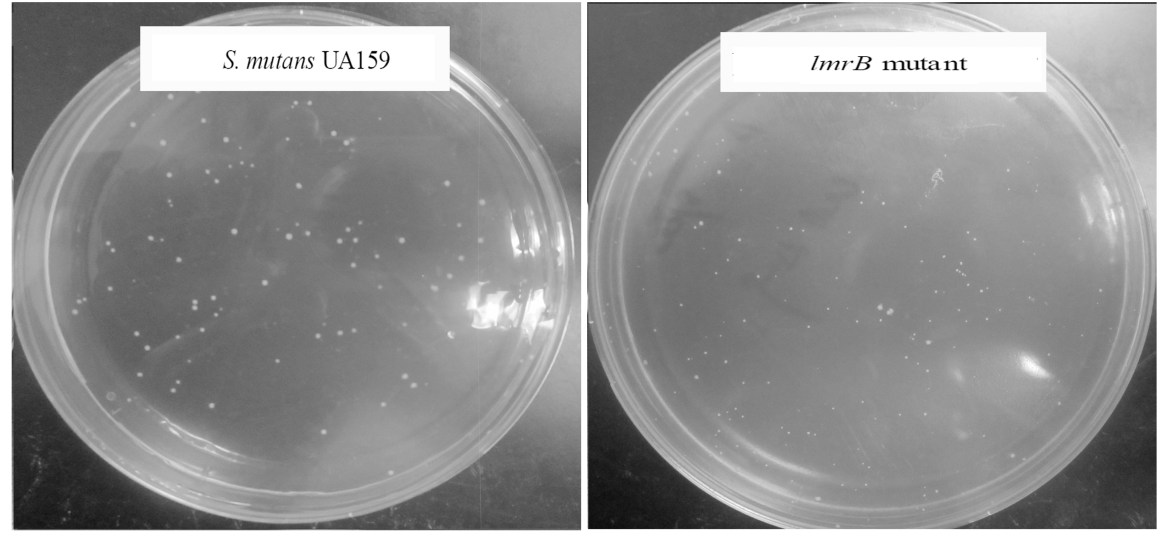


Figure S3 Quantitative RT-PCR verification of the relative expression levels of randomly selected DEGs and 16S rRNA was used for normalization. The value on the bars indicated the expression level determined by microarray. Most of the selected genes showed a similar expression trend, supporting a strong level of confidence in microarray data.


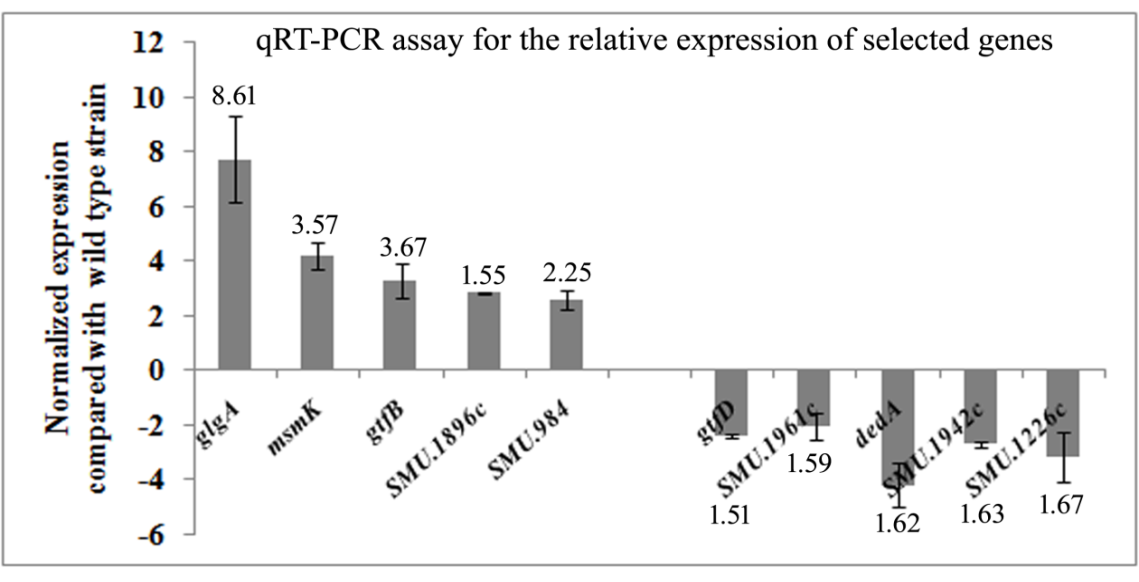


**Reference**

1. Podbielski A, Spellerberg B, Woischnik M, Pohl B, Lütticken R. 1996. Novel series of plasmid vectors for gene inactivation and expression analysis in group A streptococci (GAS). Gene. 177: 137-147.
